# Supplementary material for: Ultrahigh Performance Liquid Chromatography–Electrospray Ionization Tandem Mass Spectrometry Method for Qualitative and Quantitative Analyses of Constituents of Corydalis bungeana Turcz Extract
Source: Molecules. 2019 Sep 24;24(19):3463. doi: 10.3390/molecules24193463 (PMC6803914; doi:10.3390/molecules24193463)
Supplement: Supplementary file 1 [file molecules-24-03463-s001.pdf]

Table S1. Summary of the tested plants of *Corydalis bungeana* .

No.	Sources	Longitude	Latitude	Altitude(M)	Collection time
1	Changchun, Jilin	43.48°	125.19°	224.14	2015-08
2	Gansu	34.05°	104.35°	1100	2015-08
3	Tongjiang, Zhejiang	29.78°	119.69°	41	2015-08
4	Changzhi, Shanxi	36.19°	113.12°	931	2015-08
5	Henan	34.76°	113.75°	87	2015-08
6	Jiangsu	32.06°	118.76°	17	2015-10
7	Anguo, Hebei	38.41°	115.33°	34	2015-10
8	Yutian, Hebei	39.90°	117.73°	15	2015-10
9	Baoding, Hebei	38.87°	115.46°	25	2015-10
10	Shanxi	34.26°	108.95°	414	2016-02
11	Zhejiang	30.28°	120.15°	17	2016-02
12	Jiaocheng, Anhui	33.88°	115.77°	40	2016-02
13	Bozhou, Anhui	33.84°	115.77°	38	2016-03
14	Wenshan, Yunnan	23.40°	104.21°	1259	2016-03
15	Xinxiang, Hebei	38.07°	114.45°	84	2016-03

Table S2. Gradient elution program of mobile phase in qualitative analysis.

Time(min)	0.2% acetic acid	acetonitrile%.
0-5	70	30
5-10	69	31
10-15	69	31
15-18	67	33
18-20	67	33
20-21	66	34
21-28	66	34
28-50	48	52

Table S3. Gradient elution program of mobile phase in quantitative detection.

Time(min)	0.1% acetic acid	methanol%
0-5	70	30
5-10	69	31
10-15	69	31
15-18	67	33
18-20	67	33
20-21	66	34
21-28	66	34
28-40	55	45
40-45	55	45

Table S4. Quantitative, Qualifier ions and MS parameters of alkaloids.

Compounds	Ion pair (*m/z*)	Qualifier ion (*m/z*)	Fragmentor(V)	Collision energy (V)	polarity
acetylcorynoline	410.2→203.9	246.1	180	60	Positive
8-oxocorynoline	382.1→332.9	275.1	170	25	Positive
corynoline	368.1→289.0	177.1	180	60	Positive
tetrahydropalmatine	356.0→192.0	165.0	159	27	Positive
protopine	354.1→188.0	149.1	170	30	Positive
palmatine	352.2→336.2	308.2	158	30	Positive
columbamine	339.2→323.2	295.1	160	29	Positive
jateorhizine	338.2→294.	322.2	148	28	Positive
berberine	336.2→320.1	292.2	136	30	Positive
worenine	334.2→261.1	233.0	181	49	Positive
sanguinarine	331.9→274.0	316.9	171	40	Positive
berberrubine	322.2→307.2	279.2	160	29	Positive

coptisine	320.2→292.2	262.2	147	29	Positive
Z23	314.1→177.0	120.9	140	20	Positive

Table S5. The repeatability and stability results for fourteen reference compounds in the *C. bungeana* Turcz.

Compounds	repeatability (%)	stability (%)
acetylcorynoline	2.57	2.23
8-oxocorynoline	2.70	2.96
corynoline	2.67	4.83
tetrahydropalmatine	1.19	3.10
protopine	2.59	4.05
palmatine	2.83	1.89
columbamine	2.65	2.88
jateorhizine	2.88	4.66
berberine	1.64	3.79
worenine	2.61	1.21
sanguinarine	2.28	1.02
berberrubine	2.87	1.76
coptisine	2.87	4.41
Z23	2.27	4.58

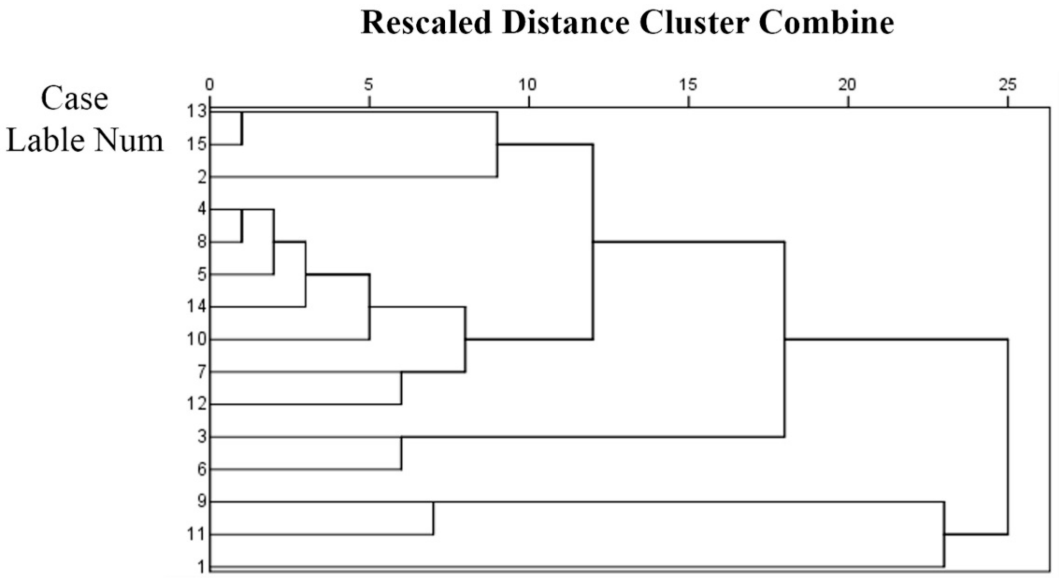

Figure S1. Cluster analysis of C. bungeana.
